# A database linking Chinese patents to China’s census firms

**DOI:** 10.1038/sdata.2018.42

**Published:** 2018-03-27

**Authors:** Zi-Lin He, Tony W. Tong, Yuchen Zhang, Wenlong He

**Affiliations:** 1 Tilburg University, 5037 AB Tilburg, Tilburg, 5000 LE, Netherlands; 2 University of Colorado, Leeds School of Business, 995 Regent Dr, Boulder, CO 80309, USA; 3 Tulane University, Freeman School of Business, 7 McAlister Dr., Golding/Woldenberg Hall, New Orleans, Louisiana 70118, USA; 4 University of International Business & Economics, 10 Huixin Dongjie, Chaoyang District, Beijing 100029, China

**Keywords:** Intellectual-property rights, Databases, Technology

## Abstract

To meet researchers’ increasing interest in the fast growing innovation activities taking place in China, we match patents filed with China’s State Intellectual Property Office to firms covered in China’s Census. China has experienced a strong growth in patent filings over the past two decades, and has since 2011 become the world’s top patent filing country. China’s Census database covers about one million unique manufacturing firms from 1998–2009, representing the broad Chinese economy. We design data parsing and pre-processing routines to clean and stem firm and assignee names, create a matching algorithm that fits with our data and maintains a balance between matching accuracy and workload of manual check, and implement a systematic manual check process to filter out false positives generated from computerized matching. Our project generates 1,113,588 matches for the Census firms, among which 849,647 patents are uniquely matched. By creating the patent-firm linked dataset, we hope to reduce duplicative effort and encourage more research to better understand China’s fast changing innovation landscape.

## Background & Summary

Patents play a central role in research on innovation. Patents provide highly detailed information on the innovation concerned, allowing researchers to conduct more fine-grained analyses of firm innovation than what is possible with aggregate R&D expenditure data or self-reported innovation activities in survey data. Patents are filed by firms of all kinds, across nearly all industries, to patent offices in many countries in the world, making it possible to build large panel datasets for longitudinal studies of innovation across firms, industries, and countries. While extremely valuable for innovation research, patent data do not come with firm identifiers that can be readily used to link to other firm-level data sources, presenting a challenge to researchers, policy makers, and business managers who seek to understand the firm-level underpinnings of innovation. In the last two decades, a number of important projects have been carried out to overcome this challenge by matching patents to the firms that own them, thereby linking patent data to the wealth of public information available on firms themselves^1–4^.

Although the resultant datasets from these matching projects have greatly expanded the number of questions that researchers are able to analyze using patent data, they almost always focused on patent offices in developed economies, notably the USPTO (United States Patent and Trademark Office) and the EPO (European Patent Office). Such a focus failed to give enough attention to the “Changing Face of Innovation^5^”—the growing prominence of innovation and patenting activities in major emerging economies, such as China^6^. China in particular has experienced a sustained, strong growth in patent filings over the past two decades. For instance, applications of invention patents, the type of patents that is most comparable across countries, to China’s State Intellectual Property Office (SIPO) increased from 14,409 in 1992 to 526,412 in 2011, overtaking the U.S. to become the world’s top patent filer^7^, a position China has since been able to secure.

To meet the increasing interest in innovation activities in China and reduce potentially duplicative matching efforts, our research team has earlier completed a project that links SIPO patents to all public firms listed on the “main board” of the Shanghai or Shenzhen Stock Exchange and their subsidiaries during 1990–2010^8^. To expand the scope of our matching effort, we conduct the current project to match SIPO patents to firms covered in China’s Annual Survey of Industrial Enterprises (ASIE). Widely dubbed the “Census data”, the ASIE data cover about 165 000 firms in 1998 to around 450 000 firms in 2009, representing the broad Chinese economy across different industries, different regions (provinces), and different types of ownership such as state-owned and non-state-owned firms, domestic and foreign-invested firms, and so forth. Because of these attractive features, we believe a database linking SIPO patents to ASIE firms will be a valuable contribution to research on Chinese patents and the growing innovation activities in China.

In this project, we first extract and pre-process all patents from the SIPO patent database, which covers all published patent applications since 1985 when the SIPO started to accept patent applications. Then we prepare and pre-process a list of all ASIE firms from 1998 to 2009. We select a matching algorithm and develop computer codes that fit with our data and maintain a balance between false positives and false negatives. We then conduct exact matching based on stem names of ASIE firms and patent assignees, and we follow the ever-matching logic for comprehensiveness and flexibility. Our automated matching program obtains 1,155,649 matches for firms in the ASIE database from 1998–2009, among which 122 897 require manual check to filter out the false positives. The automated matching program and the manual check procedure lead to a patent-firm linked dataset including 1,113,588 true matches for ASIE firms for the period 1998–2009.

## Methods

**Figure 1:**
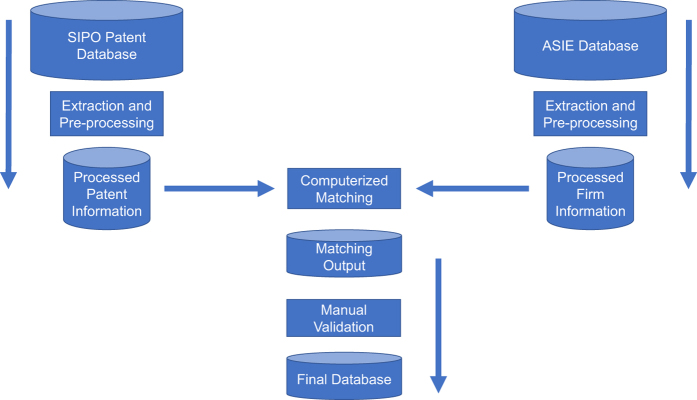
Workflow to match SIPO patents to ASIE firms and generate the final data files. This figure illustrates the workflow to match SIPO patents to ASIE firms and generate the final data files. The rectangular boxes represent the processing procedures and the cylinders the data in separate phases.

The automated matching phase, summarized in Fig. 1, is comprised of three components: data extraction, data pre-processing, and matching. Subsequent manual check is discussed in the Technical Validation section.

### Extracting SIPO patent data

We extract all patents from the SIPO patent database (DVD-ROMs, version April 2013), which covers all published patent applications since 1985 when the SIPO started to accept patent applications. In total, the database contains 2,417,903 design patents, 3,063,153 invention patents, and 2,906,059 utility model patents. Because the ASIE database covers firms from 1998–2009, we first remove patents with application dates outside the period of 1998–2009. In addition, because no individuals are included in the ASIE database and all firms covered are physically located in mainland China, we further remove the following two sets of patents before matching so as to improve computation efficiency: 1) patents assigned to individuals, and 2) patents assigned to firms with an address outside Greater China (including mainland China, Hong Kong, Macao, and Taiwan). We do not exclude SIPO patents with an assignee address in Hong Kong, Macao, and Taiwan (HMT), because as a result of increasing economic integration between the mainland and HMT, it is often difficult to discern whether an HMT firm is mainly based in the mainland or HMT. Such ambiguity is further exacerbated by the so-called “round-trip FDI” phenomenon whereby Chinese firms invest in the mainland under disguised foreign identities very often via Hong Kong^9,10^.

A patent is considered to be assigned to an individual when it meets two conditions simultaneously: 1) its inventor(s) also appear in the assignee field, and 2) the assignee field does not contain any designators of corporate form. These designators include the following Chinese strings: 股份有限责任公司, 股份有限公司, 有限责任公司, 独立行政法人, 有限总公司, 有限分公司, 总公司, 分公司, 董事会, 集团, 有限公司, 有限责任, 株式会社, 公司, 股份, 企业, 工厂, and 厂. The “and” operator is used here because firms and individual inventors may apply for patents jointly and as a result, the assignee field may contain both firm names and individual inventor names, and such patents should not be removed from the matching process. We use a long list of designators of corporate form to avoid incorrectly removing any patents that may be subsequently matched to firms in the ASIE database. Moreover, in case of 工厂 or 厂, we also require the length of the assignee to be shorter than four Chinese characters as well; this is because although some Chinese individuals may have 工厂 or 厂 in their names, Chinese individuals’ names typically have only three or two characters.

Similar sufficient conditions are applied to identify and remove patents assigned to firms with an address outside Greater China: 1) the assignee country code is outside Greater China, and 2) the assignee is a firm, i.e., the assignee name contains one of the above designators of corporate form. It is crucial to point out that since assignee address and country code are available only for the first assignee (in case of multiple assignees), we are careful to not remove any patents that have two or more assignees in the assignee field.

After removing the above three sets of patents that are irrelevant to our matching, we duplicate patents of multiple assignees into multiple records, one for each assignee. Such records have the same assignee field but different focal assignees for matching. Put another way, we assume such a patent to be equally owned by all of the assignees, and match them one by one, following He *et al.*^8^. To accelerate computerized matching, we format all patent records into nine tab-delimited text files, three for each type of patents, so that we run nine parallel threads at the same time. In total, there are 1,553,140 unique patent records to be matched with ASIE firms 1998-2009, including 468,972 unique design patents, 536,956 unique invention patents, and 547,212 unique utility model patents. Because there are important differences among these three types of SIPO patents in terms of subject matter, examination procedure, patenting and maintenance costs, maximum possible protection period, and so forth, we match them separately to ASIE firms so as to generate three separate sets of firm-patent matches.

### Extracting ASIE firm data

In this section, we first provide a description of the ASIE database, and then we document how we prepare the list of ASIE firms.

### About ASIE

The ASIE is a nationwide mandatory census administered annually by the National Bureau of Statistics (NBS) of China to firms in mining industries (6 two-digit industries from 06 to 11), manufacturing industries (30 two-digit industries from 13 to 37 and from 39-43), and utility industries (3 two-digit industries from 44 to 46). Therefore, the ASIE consists of mostly manufacturing firms. The ASIE database is also referred to as the Annual Industrial Survey Database^11,12^, the Annual Census on Industrial Enterprises^13,14^, the China Industry Census^15^, the Annual Survey of Industrial Firms^16^, or some other variants. The ASIE provides data for NBS to compute China’s GDP^17^, and the aggregated information is published in the official *China Statistical Yearbook*^10^. It contains a large number of firms distributed across different industries and throughout the country’s 31 provinces, autonomous regions, and province-equivalent municipalities (Beijing, Shanghai, Tianjin, and Chongqing). These firms account for around 95% of total Chinese industrial output and 98% of total Chinese industrial exports^18,19^. Table 1 reports the number of firms covered in our database by province and year. As the table shows, the total number of firms in our database for each year is consistent with the corresponding figure reported by NBS in the official *Yearbook* and that reported by a data vendor HuaMei Information.

Prior to 1998, NBS collected information only from state-owned firms and collectively-owned firms^14,20^. After 1998, the ASIE database covered all state-owned firms, and non-stated-owned firms—including privately-owned firms and foreign-invested firms—with annual sales of 5 million RMB (about 600–700 thousand U.S. dollars) or above^10,21^. To ensure consistency in firm coverage, data prior to 1998 are often not used in recent research^10–15^. Year 2011 witnessed another major change: NBS raised the threshold for inclusion for all firms from 5 million to 20 million RMB of total sales^14^. Since we only cover the ASIE data 1998–2009, our project is not affected by this change; in fact, post-2009 ASIE data have not been made available by NBS for academic research.

Despite the threshold of 5 million RMB of total sales (such firms are often referred to as “above scale” firms, where “scale” means the threshold defined by NBS), most ASIE firms are in fact rather small. For instance, among 162,885 firms contained in the 2000 ASIE, 7,983 were classified by NBS as large firms (4.9%), 13,741 as medium-size firms (8.4%), and 141,161 as small firms (86.7%) (see www.stats.gov.cn/tjsj/ndsj/2001c/m1303c.htm); among 336,768 firms contained in the 2007 ASIE, 2,910 were classified as large firms (0.9%), 33,596 as medium-size firms (10.0%), and 300,262 as small firms (89.2%) (see www.stats.gov.cn/tjsj/ndsj/2008/indexch.htm). It is useful to note that the NBS definition of large- and medium-size enterprises (LMEs) among these “above scale” firms varied over time and across industries^22,23^.

The ASIE database contains rich firm-level demographic, operational and financial information, including company name and address, year of founding, ownership details, 4-digit industry classifications, three main products in order of relative importance, number of employees, total production output, total output accounted for by new products, total sales, exports, profits, total assets, intangible assets, capital investment and depreciation, expenditure on R&D and advertising (available for selected years), total salary expenditure, employee training expenditure, and so forth^12,15^. In total, more than 100 variables in the firms’ main accounting statements (balance sheet, income statement, and statement of cash flows) are available^21^, making the ASIE the most comprehensive and detailed database of domestic and foreign firms operating in China. Moreover, each firm in the database has a unique firm identifier, known as the legal person code, which allows researchers to assemble a panel dataset by linking together the firm’s yearly observations^10–12,15,24,25^. However, it is not so unusual that a firm with exactly the same name and address acquires a different identifier due to ownership changes as a result of restructuring, joint ventures, mergers and acquisitions, and so forth^10,11^. Moreover, firms sometimes omit this field in their survey reports.

The information reported by ASIE firms to NBS should be fairly reliable for several reasons. First, firms are required by law to cooperate with surveys conducted by NBS^10^, and they do not have clear incentives to misreport because the information reported to NBS cannot be used against them by other government agencies such as tax authorities^17^. Second, NBS has endeavored to maintain consistency and reliability in data collection^26^. NBS has a Statistics Bureau in each county, with an office dedicated to collecting, maintaining, and reporting economic data. All the “above scale” firms have formally designated personnel who work with the Statistics Bureau to ensure that the reported data are reliable and consistent with NBS formats and definitions.

As a result, it is widely believed that the ASIE data are largely accurate and internally consistent for solid empirical work^27^. Research based on the ASIE data has been published in leading journals in different fields, including *Academy of Management Journal*^12^, *Administrative Science Quarterly*^14,28^, *American Economic Review*^29^, *American Journal of Sociology*^30^, *Economic Journal*^17^, *Journal of Economics & Management Strategy*^16^, *Journal of International Business Studies*^26,31^, *Quarterly Journal of Economics*^32^, *Review of Economics and Statistics*^15,33^, *Strategic Management Journal*^10,11,34^, and so forth.

For innovation scholars, two fields contained in the ASIE database are particularly attractive: R&D expenditure and new product sales. However, both fields can be said to suffer from serious limitations. Other than the usual critique that R&D expenditure is too coarse an indicator of innovation, data on R&D expenditure are only available from 2005-2007 in the ASIE and contain many missing values^35^. Similarly, information on new product sales is not consistently available across all firms and for all years. In addition, the definition of “new product” seems to vary across different years and across different studies. Because of these limitations, relying on ASIE data alone limits the scope and type of questions that innovation scholars can address, as well as the level of granularity of the empirical evidence that can be produced. As a result, several studies have attempted to match ASIE firms to SIPO patents to better examine these firms’ innovation activities. In the section “Comparison with prior studies” later, we will compare our matching methodology and outcome with those in prior studies.

### Preparing the list of ASIE firms

We face several challenges when compiling the list of ASIE firms for matching with patent data. First, missing firm identifiers (legal person codes) and names are common in the 2009 ASIE data we had access to. Among 448,741 entries of the 2009 ASIE, 142,963 have firm identifier missing and 136,105 have firm name missing. For firms with missing identifiers, we search their names in earlier editions of the ASIE to find the same firm name, retrieve that firm’s identifier, and use it for the year 2009. If the same firm name is found in multiple past years, the identifier for the year that is closest to 2009 will be used. A similar procedure is implemented to replace missing firm names wherever possible. Sporadic cases of missing firm identifiers and names in other years of the ASIE are dealt with in the same way.

Another issue is that there are obvious errors in some firm identifiers and names. For instance, we see strange firm identifiers such as 1 and 2 (too short to be a valid identifier), and ]15174142 (“]” is an obvious error). We also see problematic firm names such as 鄂鄂州市隆昌合金钢有限责任公司 (the second 鄂 is redundant perhaps as a data entry error), S试笫星旆嵋�铣 (this firm name is completely messed up), and 6673896 (this may be a telephone number rather than a firm’s name). Firm names like 海尔集团公司(本市) and 上海机床厂有限公司(本部) also pose problems because such content within brackets is likely to compromise matching accuracy. Spot checks by eyeballing firm names suggest that such problem cases only make up a very small minority in the vast ASIE database. Moreover, after some initial exploration, we realize that it would not be economical for us to spot and rectify each and every case in a systematic and exhaustive manner given the sheer size of the ASIE database. We therefore decide not to do anything with these obviously or potentially wrong firm identifiers and names. That said, the fact that we do “ever matching” instead of “contemporaneous matching” (more details in the section “Matching ASIE firms to patent assignees”) should help minimize miss of true matches due to incorrect ASIE firm names: this is because as long as the firm has its correctly-spelled name appearing once in the ASIE database from 1998–2009, its entire patent portfolio for the period will be captured and matched. Indeed, in two different samples each with 2,000 ASIE firm names randomly drawn from the ASIE population, we only find seven wrong firm names (0.175%) that are never correct in any of the years from 1998–2009; further search using the correct names of these seven firms in the SIPO patent database shows that none of them has patent filings during this time window.

A third issue with ASIE firm names is that the same firm may appear in slightly different names in different years, yet sometimes with rather different operational and financial details, perhaps due to restructuring, mergers and acquisitions, or significant ownership changes. Moreover, a firm in the ASIE database may assume a different identifier even when its name and address remain unchanged at all. After weighing substantial costs upfront against limited potential benefits, we opt not to consolidate ASIE firms before matching. Instead, matching is done for each unique firm identifier-name combination. To give an example, firm identifier 163578771 is linked to 海信集团 in some years but to 海信集团有限公司 in other years of the ASIE. Because we do not consolidate ASIE firm names and both 海信集团 and 海信集团有限公司 have the same stem name, which is 海信 (see the section “Pre-processing patent assignee names and ASIE firm names” for more details about stem names), the same set of patents would be matched to the firm identifier-name combination 163578771--海信集团 and to 163578771--海信集团有限公司 as well. The same applies to unique firm identifier-name combinations like 192352181--深圳市华为技术有限公司 and 192203821—深圳市华为技术有限公司, in which case two different firm identifiers are linked to exactly the same firm name. Admittedly, implementing this approach may lead to considerable duplicate matches, which we will assess in the section “Comparison with prior studies”, but one advantage of this approach is that researchers using our matched patent data can consolidate ASIE firms in different ways according to their own specific research needs, and then the matched patents can be consolidated accordingly.

In the end, we arrive at 947,166 unique firm identifier-name combinations, among which 1,705 have missing identifiers. Then, we feed the patent data and the list of ASIE firms to our matching program for pre-processing routines (see the section “Pre-processing patent assignee names and ASIE firm names”) and then computerized matching (see the section “Matching ASIE firms to patent assignee”), followed by manual check of computer outputted matches (see the section “Conducting manual check” under Technical Validation). We note that while we divide all patent data into nine different files to run nine parallel threads at the same time, a single file of ASIE firm names is fed into each thread.

### Pre-processing patent assignee names and ASIE firm names

Before matching patent assignee names and ASIE firm names, a set of pre-processing routines are implemented to clean and standardize names:

1. We trim all symbols and punctuation marks that are not letters, characters, or numbers. These include hyphen, parentheses, 《, apostrophe, comma, bar mark, etc. The content inside the parentheses, which often provides discriminating information, is kept. We make sure to remove both half-width and full-width symbols such as & and &, and both half-width and full-width punctuation marks such as ? and ?. We compile a list of symbols and punctuation marks that could cause problems in matching based on the following sources: UTF-8 Chinese symbols, ASCII English symbols, Chinese punctuation marks from https://zh.wikipedia.org/wiki/标点符号, and ASCII English punctuation marks.
2. We convert all full-width letters and numbers into half-width ones.
3. We convert Chinese numbers into Arabic numbers. Note that after implementing routine #2, all full width Arabic numbers (0, 1, 2, …,9) have already been converted into half-width ones (0, 1, 2, …, 9). Special care is needed to deal with intricate situations because firm or assignee names like 青岛四方机车车辆厂 contains one Chinese number 四, but it does not make much sense to convert 四 into 4 in this case, as 四 here does not mean anything strictly numerical. (With careful and systematic checks of both ASIE firm names and patent assignee names, we confirm that the complex form of Chinese numbers (壹, 贰, 叁, 肆, 伍, 陆, 柒, 捌, 玖, 拾) does not pose a threat to our matching.) Specifically, we sequentially implement the following steps:
4. For three- or more digit numbers, convert 零/〇, 一, 二, …, 九 into 0, 1, 2, …, 9. For example, 中国人民解放军第三五零三工厂 will become中国人民解放军第 3503工厂.
5. For two-digit numbers, convert [一…九] 十 [一…九] into 11…99, and convert 九九 into 11, …, 99.
6. For one-digit numbers, convert 第[一…十] into 第 [1…10].
7. We remove various designators of corporate form to obtain the so-called stem names. A set of such designators is the so-called stemming list, which includes 股份有限责任公司, 股份有限公司, 有限责任公司, 独立行政法人, 有限总公司, 有限分公司, 总公司, 分公司, 董事会, 集团, 有限公司, 有限责任, 株式会社 公司, 股份, 企业, 工厂, 厂. (The same list is used to determine whether a patent is an individual patent or is assigned to a firm with an address outside Greater China in the section “Extracting SIPO patent data” earlier). Such designators appear in numerous names and should be removed before matching, because they provide little information to distinguish two names.

A few examples may help make sense of these pre-processing routines. Suppose we start with name TCL-罗格朗国际电工(惠州)有限公司, it will become TCL罗格朗国际电工惠州有限公司 with routine #1, become TCL罗格朗国际电工惠州有限公司 with routine #2, no change with routine #3 as it does not contain any Chinese numbers, and then it will be stemmed into TCL罗格朗国际电工惠州. To give another example, with these four pre-processing routines, both patent assignee name 天水二一三机床电器厂 and ASIE firm name 天水２１３机床电器厂 will be standardized and stemmed into 天水213机床电器, so that the correct patents and firms will be matched. Likewise, both patent assignee name 蓝星成都六九一四电子设备厂 and ASIE firm name 蓝星成都６９１４电子设备厂 will become 蓝星成都6914电子设备, so that the corresponding true matches will be captured.

### Matching ASIE firms to patent assignees

To choose an appropriate matching algorithm and design an efficient matching architecture, we need to make several important decisions, which are informed by careful tradeoffs between false positives (a name pair matched up by the computer program in fact refers to two different entities) and false negatives (a name pair not matched up by the computer program in fact refers to the same entity), and between matching accuracy and workload of manual check (to be described later). It can be seen below that we approach each decision based on careful and iterative calibration, often with rounds of trial matching between 100,000 randomly selected patents and one year of ASIE firms’ data.

A matter of first order importance is the choice between approximate matching and exact matching. Approximate matching calculates a similarity score between two name strings and declares that a match is found when this score is above some threshold, whereas exact matching only identifies a pair of identical stem names. Approximate matching, however, comes at a price because it increases the probability of generating false positives, which have to be eliminated by subsequent manual check. Eliminating such false matches by hand requires extensive time and manpower. In He *et al.*’s^8^ matching project for listed firms that generated 222,651 matches using approximate matching, the subsequent manual check took about one month by three groups of research assistants working full time, plus two weeks by one of the authors to triangulate different manual check results. Based on several trial runs, this second project, which involves a much larger number of firms, is expected to generate about one million matches, making the time and manpower required for manual check impractical to handle. Another consideration is availability of computing power: while the average processing time is about 0.2–0.3 s when using exact matching to compare one patent assignee name against about 1 million entries in the master list of ASIE firm names, it is about 6–8 s when using approximate matching. Thus, to generate the estimated number of one million potential matches, exact matching would require a total of 83 computer hours, and approximate matching 2,499 h, outstripping the computing resources available to us. We therefore adopt exact matching based on stem names for this project.

A key concern of exact matching is loss of true matches insofar as approximate matching can capture a pair of slightly different names that in fact refer to the same entity. Our assessment is that such loss of true matches should be very limited. Among 191,325 true matches reported in He *et al.*^8^, only 3,098 (1.6%) were captured due to the additional power of approximate matching; the remaining true matches would have been captured using exact matching or by applying the “strict substring condition.” Assuming similar data structure for ASIE firms and considering the high cost of conducting approximate matching and manual check for about one million potential matches, we deem 1.6% loss of true matches acceptable. With two rounds of trial matching, we further confirm that the expected loss of true matches for ASIE firms due to exact matching rather than approximate matching is around 1.6%.

Following He *et al.*^8^, we supplement exact matching by adding the condition of “left-aligned strict substring” to minimize loss of true matches. A case of left-aligned strict substring is where the stem ASIE firm name is a strict substring of the stem assignee name from the left. Table 2 shows that such name pairs are likely to be true matches, as the patent assignee is often a division, factory, or branch office of the ASIE firm. However, this is not always the case, and thus (some of) such name pairs have to be manually checked to verify they are indeed true matches (see the section Technical Validation below for details). A natural question, however, is whether the strict substring condition should be allowed from both sides; in other words, whether the “left-aligned” restriction should be lifted to capture potentially more true matches. After multiple rounds of trial matching, we establish that without the “left-aligned” restriction, the number of computer generated matches that require manual check would increase by three to four times, yet a predominant majority of these additional matches are false matches. This is in fact consistent with the convention of Chinese organization names where the largest entity comes first from the left, followed by the division, branch, or subsidiary name. We therefore opt to keep the “left-aligned” restriction.

Another important decision is the choice between “ever-match” and “contemporaneous match”^3^. While ever-match only requires the ASIE firm name and the patent assignee name to be matched, irrespective of in which year during 1998-2009 the firm appears in the ASIE database or the patent is filed with the SIPO, contemporaneous match adds the restriction that the ASIE firm year and the patent application year must coincide. We decide to follow the ever-match logic primarily due to a common challenge in name matching: misspellings, name variations, and name changes. As noted above in the section “Preparing the list of ASIE firms” earlier, errors in ASIE firm names are not that rare. One key advantage of doing ever-match is that as long as the firm name is correctly recorded in one year of the ASIE, all relevant patents will be found and matched. Ever-match is also necessary to account for name variations or name changes. For example, 深圳市中兴通讯股份有限公司 changes its name to 中兴通讯股份有限公司 in the ASIE database since 2004, yet in the SIPO database there are many patents under the new name before 2004, and also many patents under the old name after 2004. Contemporaneous match would greatly undercount the company’s patents in this case, as name changes in these two databases are not contemporaneous or synchronized. Another important advantage is that, given that firms join or leave the ASIE database when they meet or fail to meet the “above scale” threshold of 5 million RMB total sales, ever-match allows researchers to track a firm’s patent portfolio over time even though the firm is absent in the ASIE during the intervening years. Ever-match also provides important flexibility post matching: should researchers prefer contemporaneous match, it could be easily achieved by imposing on the outcomes of ever-match the restriction of “ASIE firm year=patent application year”.

To summarize, after pre-processing ASIE firm names and patent assignee names, we conduct exact matching based on stem names, which is supplemented with strict substring matching to limit loss of potentially true matches, and we follow the ever-match logic for comprehensiveness and flexibility. With several final rounds of trial matching, we find that our matching logic is simple, clear, robust, and well-performing, doing what is designed to do.

### Code availability

The final matching program is implemented in Perl (V5.14.2) built for MSWin32-X64. The operating system is the Windows 7 enterprise edition. This program requires Encode::CN, a module that can be found at “search.CPAN.org”. By running nine threads at the same time, the computerized matching can be completed in less than four days. The Perl pseudocode is included below.[Boxed-text bx1]

## Data Records

The datasets are available from the Harvard Dataverse repository (Data Citation 1), under “Matching SIPO patents to firms in the Annual Survey of Industrial Enterprises (ASIE) of China’s National Bureau of Statistics.” These datasets contain the matched SIPO patents for all ASIE firms between 1998 and 2009.

All three data files are plain ASCII text files, as requested by the Dataverse. The three files cover Matched Design Patents, Matched Invention Patents, and Matched Utility Model Patents, respectively. In all files, each row is a matched pair between a SIPO patent and an ASIE firm. Each column is an information field obtained from the ASIE firm database or SIPO patent database. A list of variable names (including Chinese translations) and definitions appears in Table 3.

## Technical Validation

We first implemented a manual check procedure to identify false positives. We also compared our matched results with prior studies using the same data sources.

### Conducting manual check

Manual check provides researchers with additional information to more accurately identify a potentially matched name pair. However, manual check costs enormous efforts and resources in searching for information and processing the information for a more confident decision. For this project, the automated matching program generates 1,155,649 matches in total, among which 1,010,471 are based on exact matching and 145,178 are based on left-aligned strict substring matching (see Table 4). Obviously, manually checking all the name pairs outputted by our computer program is not feasible or necessary. We therefore create a set of heuristics to isolate those name pairs (one ASIE firm name and one patent assignee name) that require manual check. Specifically, we make a heuristic-based program to go through all computer reported matches and mark whether or not a name pair needs manual check.

First, when the assignee name in the name pair ends with 大学, 学院, 学校, 中学, or 小学, we deem it a case of false match and manual check not necessary. This is because such assignee names denote educational institutions, not profit-oriented industrial firms in the ASIE.

Second, when the ASIE firm name is fuzzy, showing ambiguous semantic meaning, we also mark such name pairs false matches and manual check not necessary. Table 5 gives all of the 36 such “fuzzy” names we identify from the whole list of ASIE firms. Matching for such names is meaningless and manual check cannot avail the situation.

Third, when the ASIE firm and the patent assignee have the same full name or when the firm’s full name is a strict substring of the patent assignee’s full name from the left, we mark the name pair a case of true match and manual check not necessary. (Note that in automated matching described earlier, this left-aligned strict substring rule is applied to stem names). This is because under these two conditions, the chance of having false positives is virtually zero.

Then, for the remaining name pairs, we distinguish between cases of same stem names and cases of different stem names: while the former is based on exact matching, the latter is based on left-aligned strict substring matching. For cases of same stem names, we deem those with three or more characters (i.e., the length of stem names >=3) true matches and manual check not necessary, whereas all cases of stem name length shorter than 3 require manual check. Cases of different stem names generally require manual check: as explained above and shown in Table 2, the stem firm name being a strict substring of the stem assignee name from the left is no guarantee for true matches. Nevertheless, we isolate one type of cases which we can safely deem false matches without manual check: when the ASIE name has 大学, 学院, 学校, 中学, or 小学 anywhere in the name. The logic is that such ASIE names must refer to factories or companies affiliated to an educational institution, which has been common in China; therefore the stem names must fully match if they refer to the same entity. One example is the name pair of ASIE firm 上海交通大学附属工厂 (stem name 上海交通大学附属 vs. patent assignee 上海交通大学附属第一人民医院 (stem name 上海交通大学附属第一人民医院): they are obviously two different units under 上海交通大学. Note that the set of screens described above is carefully designed based on the specific features of this matching project; if a different matching algorithm is used or if matching is directed towards a different firm database than the ASIE, these screens would need to be adapted accordingly.

As a result of the above procedures, we isolate 122,897 matches from the automated matching program that require manual check. These 122,897 matches are collapsed into 9,703 unique name pairs for manual check, which is carried out based on the following protocol or steps:

1. The first three authors did manual check for the same set of randomly selected 100 name pairs. By comparing the three sets of results, we discussed the potential reasons for differences and developed a manual check guideline, summarizing three rules:
2. Accept as true match when a) the ASIE firm and the patent assignee are the same entity, b) one is part of the other, or c) one is a predecessor or successor of the other.
3. Accept as true match when no information is available to clearly separate the two entities, e.g., when information is hardly available for one of the two names.
4. Accept as true match if the ASIE firm and the patent assignees in a name pair are located in the same province or city whose name appears in the firm and assignee names, and the two names are very similar (suggesting that one may be part of the other, but there is no unambiguous information indicating so).
5. We hired six senior undergraduate students from a top university in China as research assistants. We gave them a workshop to explain the manual check procedure. Each student was subsequently given a same set of 50 name pairs for manual check. A meeting was then held to discuss and sort out any differences in their manual check outcomes.
6. These six RAs were divided into two teams to do manual check for all 9,703 unique name pairs by following the above protocol. Within each team, each RA dealt with around 3,200 name pairs. With this procedure, two manual check results were independently obtained for each name pair. These two sets of manual check had a Cohen’s Kappa of 0.77, indicating sufficient agreement between the coders.
7. The first author did the final check. If a name pair received unanimous “yes—this is a case of true match” or unanimous “no—this is not a case of true match”, it would be accepted as such. Otherwise, additional search was conducted to make a final decision.

By the end of this process, out of 122,897 computer generated matches that require manual check, we could confirm that 90,860 are true matches. Table 4 shows the breakdown of manual check results for each of the three types of patents.

Note that in the matched data files available on the Harvard Dataverse, we include not only the true matches but also the false matches that we identify in our project (indicated by the “True match flag”), and we also include a column to mark for each computer outputted name pair whether manual check is needed (indicated by “Manual check flag”). With these two flags, users of our dataset can easily trace which screen or rule is invoked, why we deem a particular name pair true or false match, and why manual check is or is not needed to establish that. We aim to be as thorough and as transparent as possible in documenting our matching project, so that future scholars can use the detailed information reported for potential replication, verification, and extension.

We assess the extent to which our matched results are improved by combining computerized matching and manual check compared with computerized matching alone. Specifically, we assess degrees of contamination and omission of two alternative, naïve approaches using the information reported in Table 4: 1) regard all computer-generated name pairs as true matches, which leads to contamination by false positives, and 2) regard all name pairs requiring manual check as false matches, which leads to omission of true positives. As shown in Table 6, the assessment indicates that our two-pronged matching approach provides non-negligible improvement over the alternative of only relying on computerized matching.

We also evaluate the reliability of our matching approach by comparing the current matching output against the SIPO-listed firm matching output reported in He *et al.*^8^. We first isolate all firms that have the *identical* full name, and observation year, in both the listed firm database and the ASIE firm database to make sure that the same group of firms is used for evaluation. Next, for this group of firms shared between the two databases, we locate their matched patents in the two respective matching projects, for each year from 1998 to 2009 and for all three types of patents. Then, for each year from 1998 to 2009, we aggregate patent level information to the firm level and calculate the correlation of firm patent counts between the two matching projects, in order to assess consistency in the matching outputs. We find that across the three types of patents, the mean of correlations is 0.97, indicating a high level of consistency in the matching outputs between the two projects.

### Comparison with prior studies

Our computer matching program obtains 1,155,649 matches for firms in the ASIE database 1998-2009, among which 1,113,588 are “true” matches. The distribution among the three different types of patents can be found in Table 7. Moreover, as explained earlier, significant duplicate matches are expected since we do not consolidate ASIE firm names. The table shows that among 1,113,588 true matches, 849,647 patents are uniquely matched. Recall that a total of 1,553,140 unique patent records are fed into our matching program (see the section “Extracting SIPO patent data”)—this means that around 55% of the patents in the pool are matched to ASIE firms, confirming the broad coverage of Chinese firms in the ASIE database. The table also indicates that duplicate matches account for less than a quarter of the matched patents. As suggested earlier, users of our dataset may choose different ways to remove duplicate matches based on their preferred approach to consolidating ASIE firm names.

We are aware of several prior studies that combined SIPO patent data with ASIE firm data in their research. In a first study, Hu and Jefferson^23^ use firms’ self-reported number of patent applications to examine what is behind China’s patent explosion, based on a sample of approximately 20,000 large and medium-sized enterprises (LMEs) drawn from the ASIE database. However, self-reported patent numbers are likely subject to problems of unreliable recalls or “retrospective response bias”^18,36^; moreover, no distinction is made among the three different types of Chinese patents. In another, more recent study, Hu and his colleagues^37^ use a matched dataset linking SIPO patents to the LMEs in the ASIE database from 2007 to 2011, to examine factors driving Chinese firms’ fast growth in patent filings. They indicate that the matching was conducted by the SIPO and NBS using the firm's legal person code (i.e., the ASIE firm identifier), rather than the name. However, as we have shown in the section “About ASIE” earlier, LMEs are only a rather small subset of the enterprises included in the ASIE database. Besides covering a shorter yet more recent period of time (2007-2011), NBS/SIPO are not making public the matched data nor the documentation of the matching approach. As a result, scholars are not able to validate the matched data independently; for instance, users may want to know how the issue of one firm acquiring different legal person codes due to ownership changes is taken into account when matching is done based on legal person codes. By contrast, our matching project includes all ASIE firms (large and small) and focuses on a different time window (1998-2009). We are also making public the matched data and the documentation of our matching approach, for validation, replication and extension by other scholars to improve on our work.

Four other studies conducted the matching of SIPO patents to ASIE firms. In a first study, Eberhardt *et al.*^18^ match SIPO patents contained in the PATSTAT database to ASIE firms 1999-2006. Their matching is “indirect” in that they use BvD’s Oriana database to bridge firm names that appear in the ASIE (Chinese characters only) and those appearing in PATSTAT (pinyin transcription, English translation, or a mix of both). This indirect approach raises several concerns. First, BvD has very limited coverage of Chinese firms. In fact, the authors acknowledge that “The Oriana version available to us contains firm-level data for about 23,000 Chinese firms for the period 2000-2005” (p. 10), which is a small subset of the ASIE data that contain about 200,000 firms each year during this period. Second, while PATSTAT contains most of the SIPO invention patents and utility model patents, its coverage of SIPO design patents is very poor. Third, names in PATSTAT and Oriana might differ substantially depending on whether they are transcribed using pinyin or (partly) translated into English; this added complication might greatly reduce matching accuracy and efficiency.

In a second study, Dang and Motohashi^38^ match ASIE firms with SIPO patents. Their matching of all the observations in the ASIE data (1998-2008) with patent data by names of companies leads to 126,386 invention patent applications from 12,208 firms. The authors report a table showing the distribution of matched patents by year, but do not provide details about how their matching approach is designed and implemented.

In a third study, Chen *et al.*^25^ match ASIE data with SIPO patent data to examine how privatization affects firm innovation, but no explanation whatsoever is given regarding how the two types of data are linked together.

In a fourth study, Xie and Zhang^39^ conduct a “general purpose” matching like our project, and they focus on the same time window from 1998-2009. While the authors report that their matching is also based on firm names, they do not provide much detail about how their matching approach (e.g., contemporaneous or ever matching; left-aligned strict substring matching) is designed and implemented. In addition, there is no mention about detailed matching criteria or manual check.

None of the four projects makes public their matched data, making it impossible to validate their data or analyze their data in a fine-grained fashion. Table 8 provides a comparison of our number of matched patents with those reported in the four abovementioned studies. As the last two columns in the table show, the number of unique patents matched in our project is much larger than the corresponding numbers reported in the first three previous matching efforts. We see three major reasons for the possibly superior performance of our matching approach. First, in contrast to Eberhardt *et al.*^18^ whose matching approach is indirect, we match firm names in the ASIE database to assignee names in the SIPO database directly, both of which are in Chinese characters. Second, we implement ever-matching such that as long as a firm appears in any one of the twelve years (1998-2009) of the ASIE database, all of its patents during the period will be captured and matched. This approach improves on that in Dang and Motohashi^38^ and Chen *et al.*^25^, who implement contemporaneous matching by first isolating a balanced panel of firms from the ASIE database. Finally, we design and implement a systematic, iterative approach that includes multiple calibration procedures and careful manual check.

Our number of unique granted patents matched (737,834) is slightly smaller than the number of granted patents (749,691) reported in Xie and Zhang^39^. We offer several comments on the difference between the two studies. First, while our count is based on unique patents matched, it is unclear whether they count the same way. Second, our patent data (DVD-ROMs, version April 2013) are one year earlier than their data, which include all patents approved as of May 1, 2014. Of course, only invention patents will be affected in this case, since design patents and utility model patents are almost 100% granted at the SIPO. Third, the two studies differ in the time window used for forming the base sample of patents for matching. While we limit the base sample to patent applications filed by domestic firms and organizations from 1998-2009, they indicate that their base patent sample includes patents granted to domestic firms from 1985-2009. Fourth, a question arises why we include in the base sample patent applications filed by not only domestic firms but also domestic “organizations”. The motivation is to avoid missing potential true matches for ASIE firms that do not have a typical firm designator in their names (see the section “Extracting SIPO patent data” for a list of such designators in Chinese characters). As we survey the ASIE database, we find many such cases: some firms are labelled as a research institute (e.g., 广州市利硕分离科学研究所), a technology testing center (e.g., 博世呼伦贝尔汽车测试技术中心), a printing house (e.g., 重庆西南铝鑫鸿印刷所), a coal mine (e.g., 宝丰县周庄镇玉厚煤矿), and many more. In total, among 947,166 unique ASIE firm identifier-name combinations fed into our matching program, 32,836 do not contain a typical firm designator in their names, yet some of them do file patent applications.

Table 9 reports the distribution of our matched patents, by year and type of patents.

Finally, we note that some scholars examine foreign multinational firms’ patenting activities at the SIPO. For example, Holmes *et al.*^40^ match SIPO invention patents during 2005-2010 to 114 large foreign multinational firms doing business in China, and focus on examining the 10,184 SIPO patents jointly owned by foreign multinationals (often through their affiliates in China) and a domestic Chinese partner. Studies such as this are not directly comparable with our study given the different focus on patenting firms that seek patents at the SIPO.

### Usage notes

The data files hosted on the Harvard Dataverse are plain text files with csv formatting. Software that supports csv format can be used to import and further process these files. For example, Microsoft Excel 2016, Stata, SPSS, SAS, R, or frequently used programming languages (e.g., Python, Perl, C++, etc.) are suitable for processing the data files. For Stata version 12 or earlier, there is a lack of support for Chinese characters. Thus, some or all Chinese characters may not be shown properly after importing; however, this should not affect the usage of the data files because numbers such as date, identification number, and other English letters will still be imported correctly.

## Additional information

**How to cite this article**: He, Z.-L. *et al.* A Database Linking Chinese Patents to China’s Census Firms. *Sci. Data* 5:180042 doi: 10.1038/sdata.2018.42 (2018).

**Publisher’s note**: Springer Nature remains neutral with regard to jurisdictional claims in published maps and institutional affiliations.

## Supplementary Material



## Figures and Tables

**Table 1:** Distribution of ASIE firms across 31 provinces, autonomous regions, and province-equivalent municipalities, 1998-2009.

Province	1998	1999	2000	2001	2002	2003	2004	2005	2006	2007	2008	2009
Anhui	3,820	3,782	3,681	3,674	3,920	4,159	4,793	5,278	6,523	8,111	11,112	14,516
Beijing	4,502	5,240	4,575	4,327	4,557	4,024	6,906	6,297	6,400	6,397	6,886	6,934
Fujian	6,103	5,548	6,006	6,554	7,474	9,211	11,953	12,396	13,755	15,178	16,898	18,743
Gansu	1,654	2,253	2,859	3,097	3,217	2,894	2,022	1,733	1,733	1,841	1,807	1,993
Guangdong	17,977	18,881	19,695	20,652	22,620	24,519	34,738	35,157	37,494	42,260	51,134	53,422
Guangxi	3,365	3,146	3,159	3,059	2,913	2,873	3,751	3,686	4,049	4,408	5,089	5,841
Guizhou	2,051	2,121	2,088	1,923	2,069	2,123	2,546	2,584	2,594	2,295	2,501	2,807
Hainan	640	578	596	573	602	620	634	616	595	488	521	500
Hebei	7,524	7,337	7,164	7,511	7,536	7,816	9,345	9,938	10,633	10,870	12,192	13,509
Henan	10,445	9,913	9,924	9,644	9,663	9,089	11,741	10,867	11,895	13,510	17,825	18,754
Heilongjiang	3,558	2,995	2,716	2,504	2,635	2,612	3,345	2,887	2,956	3,172	4,296	4,496
Hubei	7,398	6,871	6,281	6,146	6,176	6,272	6,366	6,814	7,546	8,995	11,759	14,214
Hunan	4,557	4,797	4,808	4,779	5,439	5,959	7,610	8,022	8,999	10,201	11,345	13,509
Jilin	2,845	2,837	2,768	2,606	2,622	2,343	3,451	2,774	3,249	3,984	5,151	6,133
Jiangsu	17,997	18,004	18,313	19,610	21,467	23,856	40,899	32,224	36,319	41,841	63,610	63,380
Jiangxi	3,951	3,737	3,556	3,105	3,085	3,054	4,263	4,403	5,333	6,028	6,750	7,712
Liaoning	6,250	5,806	6,018	5,693	6,018	6,844	11,458	11,509	14,754	16,556	21,124	26,276
Inner Mongolia	1,368	1,280	1,262	1,195	1,320	1,531	2,284	2,448	3,074	3,363	3,691	4,639
Ningxia	539	521	435	424	408	437	666	685	761	745	876	1,007
Qinghai	570	555	441	378	396	398	478	406	437	473	470	552
Shandong	11,443	11,432	11,721	12,149	13,508	16,226	23,916	27,540	31,936	36,145	41,927	46,671
Shanxi	3,934	3,349	3,280	3,021	3,460	3,610	5,067	4,441	4,671	4,472	4,296	4,049
Shaanxi	2,683	2,587	2,551	2,329	2,464	2,489	3,117	2,998	3,372	3,373	3,702	4,494
Shanghai	9,401	9,340	8,588	9,745	10,094	11,126	15,766	14,806	14,403	15,099	18,291	18,043
Sichuan	4,982	4,542	4,399	4,477	4,907	5,434	7,454	7,958	8,995	10,709	13,258	13,461
Tianjin	5,437	5,245	5,465	5,598	5,376	5,381	6,466	6,145	6,302	6,361	7,658	8,639
Xizang	340	328	361	363	345	324	187	195	202	98	74	90
Xinjiang	1,821	1,627	1,456	1,326	1,266	1,256	1,446	1,445	1,481	1,575	1,807	2,022
Yunnan	2,514	2,131	2,122	1,988	2,070	1,992	2,398	2,362	2,603	2,699	2,994	3,547
Zhejiang	13,450	13,274	14,552	18,549	21,869	25,508	41,369	40,277	45,688	51,604	57,739	62,271
Chongqing	1,997	1,976	2,042	2,032	2,061	2,242	2,657	2,943	3,208	3,916	5,987	6,481
Province unknown	2	0	1	0	0	0	0	1	0	0	47	36
Our total	165,118	162,033	162,883	169,031	181,557	196,222	279,092	271,835	301,960	336,767	412,817	448,741
HMI total	165,119	162,034	162,885	169,031	181,557	196,222	279,092	271,835	301,961	336,768	412,000	434,000
NBS total	165,080	162,033	162,885	171,256	181,557	196,222	276,474	271,835	301,961	336,768	426,113	434,364
This table reports the number of firms covered in our database by province (autonomous region, municipality) and year. HMI (HuaMei Information) total can be found at http://www.allmyinfo.com/data/zggyqysjk.asp; NBS total can be found at http://www.stats.gov.cn/tjsj/ndsj.												

**Table 2:** Examples of the left-aligned strict substring condition.

**ASIE firm name**	**Stem firm name**	**Patent assignee name**	**Stem assignee name**	**True match?**
贵州黄果树烟草集团公司	贵州黄果树烟草	贵州黄果树烟草集团有限责任公司贵阳烟叶购销分公司原料分厂	贵州黄果树烟草贵阳烟叶购销原料分	Yes
鞍山钢铁集团公司	鞍山钢铁	鞍山钢铁集团公司水泥厂	鞍山钢铁水泥	Yes
长飞光纤光缆有限公司	长飞光纤光缆	长飞光纤光缆（上海）有限公司	长飞光纤光缆上海	Yes
上海创开无框阳台有限公司	上海创开无框阳台	上海创开无框阳台窗有限公司	上海创开无框阳台窗	Yes
上海宝钢集团公司	上海宝钢	上海宝钢建筑工程设计研究院	上海宝钢建筑工程设计研究院	Yes
北京市北郊冷饮食品厂	北京市北郊冷饮食品	北京市北郊冷饮食品三厂	北京市北郊冷饮食品三	No
天津市自动化仪表厂	天津市自动化仪表	天津市自动化仪表七厂	天津市自动化仪表七	No
洛阳市工程机械厂	洛阳市工程机械	洛阳市工程机械设计所	洛阳市工程机械设计所	No
This table lists several exemplary inputs and outputs of left-aligned strict substring matching. ASIE firms’ full names and stem names are in the first and second columns. Patent assignees’ full names and stem names are in the third and fourth columns. The matching output (true match or not) based on stem names is in the last column.				

**Table 3:** Variable names and definitions.

**Column**	**Variable Name (Chinese Translation)**	**Definition**
A	ASIE_id (ASIE 企业代码)	Unique ID of ASIE firm
B	Fullname (ASIE 企业全名)	Full name of ASIE firm
C	Stemname (ASIE 企业全名)	Stem name of ASIE firm
D	Patent_type (专利类型)	Type of patent: d = design patent; i = invention patent; u = utility model patent
E	Serial_no (序列号)	Serial number of patent in SIPO CD-ROMs
F	Application_year (申请年份)	Patent application year
G	Assignee (申请人)	Assignee field
H	Assignee_full (申请人全名)	Full name of focal assignee to be matched
I	Assignee_stem (申请人根名)	Stem name of focal assignee to be matched
J	Manual_check (手工较验)	Manual check flag: 1 = manual check is needed; 0 = manual check is not needed
K	True_match (正确匹配)	True match flag: Yes = true match; No = false match
L	Publication_date (公开日)	Patent publication date
M	Application_date (申请日)	Patent application date
N	Primary_class (主分类号)	Primary technology class
O	Class (分类号)	All technology class(es)
P	Divisional_application (分案原申请号)	Number of prior SIPO application, if any, that focal application refers to
Q	Priority (优先权)	Priority number(s)
R	Address (地址)	Address of the first assignee
S	Patent _agency (专利代理机构)	Name of patent agency
T	Patent_attorney (代理人)	Name of patent attorney
U	Pages (页数)	Number of pages of patent application
V	Country_or_province_code (国省代码)	Country/province code of the first assignee
W	Grant (专利授权)	Grant status as of April 2013: 1 = granted; 0 = not granted
X	Grant_date (专利授权日)	Grant date as of April 2013 (NA if not granted)
This table lists the variable names and definitions of the final database.		

**Table 4:** Breakdown of results from automated matching program and manual check.

Design patents		Invention patents		Utility model patents																			
398,483		332,682		424,484																		
Exact matching		Left-aligned strict substring matching		Exact matching		Left-aligned strict substring matching		Exact matching		Left-aligned strict substring matching											
356,121		42,362		291,449		41,233		362,901		61,583										
Manual check required		Manual check not required		Manual check required		Manual check not required		Manual check required		Manual check not required		Manual check required		Manual check not required		Manual check required		Manual check not required		Manual check required		Manual check not required	
575		355,546		38,621		3,741		578		290,871		32,195		9,038		686		362,215		50,242		11,341	
True matches	False matches	True matches	False matches	True matches	False matches	True matches	False matches	True matches	False matches	True matches	False matches	True matches	False matches	True matches	False matches	True matches	False matches	True matches	False matches	True matches	False matches	True matches	False matches
551	24	355,527	19	29,022	9,599	2,150	1,591	532	46	290,094	777	22,983	9,212	3,659	5,379	605	81	361,833	382	37,167	13,075	9,465	1,876
This table reports the subtotals of automated matching results and manual checks. Total number of computer generated matches based on exact matching=356,121+291,449+362,901=1,010,471. Total number of computer generated matches based on left-aligned strict substring matching=42,362+41,233+61,583=145,178. Total number of matches that require manual check=575+38,621+578+32,195+686+50,242=122,897, among which 90,860 are true matches and 32,037 are false matches. Total number of matches that do not require manual check=355,546+3,741+290,871+9,038+362,215+11,341=1,032,752, among which 1,022,728 are true matches and 10,024 are false matches.																							

**Table 5:** List of “fuzzy” ASIE firm names.

1	印刷厂
2	建材厂
3	机械厂
4	林化厂
5	电机厂
6	电缆厂
7	食品厂
8	无线电厂
9	水电公司
10	油脂集团
11	热电公司
12	物流公司
13	电业公司
14	电力公司
15	黄金公司
16	天然食品厂
17	建筑材料厂
18	水电总公司
19	电力总公司
20	石油化工厂
21	矿业总公司
22	第三化工厂
23	有色金属公司
24	汽车零部件厂
25	油脂有限公司
26	电力工业公司
27	电力集团公司
28	电装有限公司
29	造纸有限公司
30	医药集团有限公司
31	电力有限责任公司
32	食品集团有限责任公司
33	2
34	鼎盛
35	微生物研究所
36	陶瓷有限责任公司
This table provides a full list of ASIE firm names with ambiguous semantic meaning.	

**Table 6:** Assessing degrees of contamination and omission of alternative approaches.

	Our approach:	Alternative 1:	Alternative 2:
	Combine computerized matching and manual check	Regard all computer-generated name pairs as true match	Regard all name pairs requiring manual check as false match
# of matched design patents	387,250	398,483 (degree of contamination: 2.9%)	357,677 (degree of omission: 7.6%)
# of matched invention patents	317,268	332,682 (degree of contamination: 4.9%)	293,753 (degree of omission: 7.4%)
# of matched utility model patents	409,070	424,484 (degree of contamination: 3.8%)	371,298 (degree of omission: 9.2%)
This table reports the assessment of our matching approach against alternative approaches. Degree of contamination=100×(“alternative 1” – “our approach”) / “our approach”. Degree of omission=100×(“our approach” – “alternative 2”) / “our approach”.			

**Table 7:** Assessing the extent of duplicate matches.

	Computer generated matches	Unique patents matched		TRUE matches after manual check	Unique patents matched	
	(a)	(b)	%(b/a)	(c)	(d)	%(d/c)
Design patents	398,483	300,956	75.5	387,250	291,578	75.3
Invention patents	332,682	265,338	79.8	317,268	253,628	79.9
Utility model patents	424,484	316,303	74.5	409,070	304,441	74.4
Total	1,155,649	882,597	76.4	1,113,588	849,647	76.3
This table reports the assessment of the extent of duplicate matches for the three different types of patents						

**Table 8:** Comparing our number of matched patents with that in prior studies.

Authors	ASIE period	Number of firms	Distinguish different types of patents?	Number of patents matched	Our number of unique patents matched during the same period
Eberhardt et al. ^18^ ^a^	1999-2006	about 590,000	Only invention patents are matched	44,344 (invention patents)	95,902 ^b^ (invention patents)
Dang & Motohashi ^38^	1998-2008	12,208 (panel data)	Only invention patents are matched	126,386 (invention patents)	188,773 (invention patents)
Chen et al. ^25^	1998-2007	11,631 (panel data)	Yes	50,013 (three types of patents in total)	484,359 (three types of patents in total)
Xie & Zhang ^39^	1998-2009	682,814	Yes	749,691 granted patents ^d^ (three types of patents in total)	849,647 patent applications, among which 737,834 are granted ^e^ (three types of patents in total)
This table provides a comparison of the number of matched patents in this study with the numbers reported in the four prior studies. a). Eberhardt et al^18^ match SIPO patents contained in the PATSTAT database to ASIE firms indirectly via Oriana. They acknowledge that “Oriana only contains a subset of the firms contained in the census” (p. 11) and “The Oriana version available to us contains firm-level data for about 23,000 Chinese firms for the period 2000-2005” (p. 10). b). Despite using ASIE data 1999-2006, Eberhardt et al^18^ count invention patents applied for between 1985 and 2006 (see the Appendix therein). We report here the number of matched invention patents filed between 1999 and 2006, making a conservative comparison with theirs. c). This is obtained by multiplying the number of observations (116,310) and mean number of patent applications (0.43, counting all three types of patents). d). This number is calculated by summing up the annual number of matched patents from 1998 to 2009 reported in Table 9 of Xie and Zhang ^39^. These patents are granted as of May 1, 2014. e). These patents are granted as of as of April 2013.					

**Table 9:** Distribution of matched patents, by year and type of patents.

	Design patents		Invention patents		Utility model patents		Three types in total	
	(1)		(2)		(3)		(4)	
Year	Number of matches	%	Number of matches	%	Number of matches	%	Number of matches	%
1998	9,375	2.4	1,226	0.4	5,430	1.3	16,031	1.4
1999	13,089	3.4	2,162	0.7	8,011	2	23,262	2.1
2000	15,095	3.9	3,392	1.1	9,848	2.4	28,335	2.5
2001	16,741	4.3	4,822	1.5	12,053	2.9	33,616	3
2002	22,373	5.8	9,281	2.9	16,938	4.1	48,592	4.4
2003	23,652	6.1	14,630	4.6	21,855	5.3	60,137	5.4
2004	26,986	7	18,420	5.8	23,435	5.7	68,841	6.2
2005	31,863	8.2	26,868	8.5	29,807	7.3	88,538	8
2006	41,118	10.6	38,828	12.2	40,384	9.9	120,330	10.8
2007	52,071	13.4	50,791	16	52,103	12.7	154,965	13.9
2008	60,354	15.6	65,539	20.7	76,205	18.6	202,098	18.1
2009	74,533	19.2	81,309	25.6	113,001	27.6	268,843	24.1
Total	387,250	100	317,268	100	409,070	100	1,113,588	100
This table reports the distribution of our matched patents, by year and type of patents. Columns 1 to 3 report the distribution for the three types of patents separately, and Column 4 reports the distribution for all three types of patents combined.								

## Box

01.USE Encode::CN;
02.SET $AsieDataLocation;
03.SET $PatentDataLocation;
04.SET $OutputLocation;
05.READ @AsieFirms from $AsieDataLocation;
06.READ @Patents from $PatentDataLocation;
07.FOREACH $FirmName in @AsieFirms {
08.     FOREACH $AssigneeName in @Patents {
09.         PREPROCESS $FirmName;
10.         PREPROCESS $AssigneeName;
11.         EXACTMATCH $FirmName against $AssigneeName;
12.         IF $MatchOutcome is positive THEN {
13.             WRITE $MatchOutcome to $OutputLocation;
14.         }
15.     }
16.}
